# Recent advances in the tumour biology of the GPI-anchored carcinoembryonic antigen family members *CEACAM5* and *CEACAM6*

**DOI:** 10.3747/co.2007.109

**Published:** 2007-04

**Authors:** C.H.F. Chan, C.P. Stanners

**Keywords:** Carcinoembryonic antigen, carcinoembryonic antigen–related cell adhesion molecule 6, cancer, colon cancer, tumour biology

## INTRODUCTION

On its discovery in 1965 by Gold and Freedman^1^ in the blood of patients with colorectal cancer, human carcinoembryonic antigen [*CEA* (since re-designated *CEACAM5*)] was initially thought to be a tumour-specific antigen. Although *CEACAM5* was subsequently found in normal tissues, its consistent overexpression in many cancers has made it a tumour marker widely used for patient management and a popular molecular target for novel cancer therapies.

After the cloning of *CEACAM5* cdna in 1986^2^, other *CEACAM5-*related cell adhesion molecules were also identified in humans and other mammalian species 3–5. The *CEACAM* family members are highly glycosylated proteins that belong to the immunoglobulin gene superfamily^6^. In humans, the *CEACAM* family consists of membrane-linked and secretory glycoproteins. The former are anchored to the cell surface either by a glycophosphatidyl–inositol (gpi) anchor or a transmembrane domain. The gpi-anchored members include *CEACAM5* (the original *CEA*), *CEACAM6, CEACAM7,* and *CEACAM8*^3^. Thus far, the gpi-anchored *CEACAM*s have been detected only in primates, and not in lower mammals^3^–^5^,^7^.

The enormous volume of literature describing the aberrant expression of *CEACAM5* and *CEACAM6* in various types of cancers, the prognostic values of such expression, and *CEACAM5-*targeted therapies has tended to dilute studies revealing the significant biologic functions of these antigens and their potential clinical implications. This editorial overview highlights current knowledge of the biologic functions of *CEACAM5* and *CEACAM6* in relation to tumorigenesis.

## *CEACAM5* AND *CEACAM6* IN HUMAN CANCERS

*CEACAM5* is overexpressed in cancers of the gastrointestinal tract, pancreas, liver, gallbladder, lung, breast, female reproductive system, medullary thyroid, urinary bladder, and prostate^3^,^8^–^11^. Similarly, *CEACAM6* is overexpressed in cancers of the colon, stomach, pancreas, lung, breast, and female reproductive system, and in leukemia^3^,^8^. Overall, *CEACAM5* or *CEACAM6,* or both, are overexpressed in as many as 70% of all human tumours^12^. In addition, that overexpression is often associated with poor prognosis—specifically, poor clinical outcome and reduced survival^13^–^16^.

This overwhelming correlation suggests an instrumental role for these molecules in tumorigenesis. In fact, *CEACAM5* and *CEACAM6* have a variety of tumorigenic effects on cells cultured *in vitro* and in *in vivo* model systems. Overexpression of *CEACAM5* and *CEACAM6* impedes myogenic, adipogenic, neurogenic, and colonic differentiation programs^17^–^19^, inhibits anoikis and apoptosis in colon and pancreatic cancer cells^20^–^22^, disrupts cell polarization and tissue architecture^19^, enhances liver metastasis^22^,^23^, increases chemoresistance^24^, and increases colon-tumour^25^ and lung-tumour (Chan *et al.* Higher incidence of spontaneous lung tumours in the CEABAC mice. In preparation) susceptibility in a transgenic mouse model.

This broad spectrum of tumorigenic effects arises from functions at the molecular level. *CEACAM5* and *CEACAM6* have been shown to activate integrin signalling pathways^26^,^27^. Proteins that are gpi-anchored, including *CEACAM5* and *CEACAM6,* are often localized in the membrane microdomains called “lipid rafts” 28. These rafts carry specific subsets of signalling molecules and are freely mobile on the cell membrane. Growing evidence suggests the presence of specific types of lipid rafts 28,29. *CEACAM5* and *CEACAM6* have been shown to be co-localized with integrin α5β1 in the same specific lipid rafts 30.

*CEACAM5* and *CEACAM6* function as intercellular adhesion molecules because of parallel and anti-parallel self-binding of their extracellular domains 31, and therefore small *CEACAM5-* and *CEACAM6-*containing lipid rafts can cluster together to form bigger rafts 29, thus co-clustering their associated signalling elements. This co-clustering could underlie the observed activation of downstream signalling cascades, such as the integrin signalling pathway, including elements *ILK, PI3K*, and *AKT* 26. This mode of signal activation would critically depend on the cell-surface level of *CEACAM5* and *CEACAM6.* That is, the downstream signal and consequent cellular behaviour would depend in a nonlinear threshold fashion on the concentration of *CEACAM5, CEACAM6,* or both.

## *CEACAM5* AND *CEACAM6* IN COLORECTAL CANCER

Colorectal cancers are the end result of multiple transformational events in normal epithelia. A set of neoplastic events, termed the adenoma–carcinoma sequence, was originally proposed by Vogelstein and colleagues for traditional adenomas 32. The loss of functional *APC* causes a transition from normal epithelium to aberrant crypt foci (acf), the earliest detectable tumorigenic change, followed by Kras activation (adenoma formation), loss of *SMAD2* and *SMAD4,* and *TP53* inactivation (carcinoma formation). With growing knowledge of the genetics of colorectal cancers, more gene mutations are being placed into this basic paradigm, although all the events are not necessarily present and their sequence can vary 33,34.

In contrast to the traditional adenomatous polyps, hyperplastic polyps are commonly believed not to progress to malignant lesions 35. However, in recent years, sessile serrated adenoma, serrated adenoma, and mixed polyps (a subgroup of hyperplastic lesions showing a serrated feature) have been shown to have malignant potential 36. These serrated lesions show frequent *BRAF* (a member of the *RAF* family of serine and threonine kinases) mutations and widespread dna methylation, and they have recently been considered premalignant lesions that follow the serrated pathway of neoplastic transformation as proposed by Jass and colleagues 36–38. A general inhibition of anoikis caused by mutation in a specific gene can lead to serrated polyp formation 37. Mutations in or downregulation of *hMLH1* or *MGMT* (methylguanine methyltransferase) can then lead to progression to msi-h (high level of microsatellite instability) and msi-l (low level of microsatellite instability) colorectal cancers respectively 38.

Although *CEACAM5—*and to a lesser extent *CEACAM6—*are consistently overexpressed in most colorectal cancers and have a broad range of tumorigenic effects, they have not yet been assigned to any proposed pathway. On the one hand, the overexpression of *CEACAM5* in 30%–90% of acfs suggests that this overexpression can be an early event in the adenoma–carcinoma sequence 39,40. On the other hand, *CEACAM5* overexpression in serrated polyps and its anti-apoptotic ability may suggest its involvement in the serrated pathway 41. Similarly, the overexpression of *CEACAM6* in hyperplastic polyps and traditional adenomas alike suggests that *CEACAM6* may also be involved in these neoplastic pathways 42.

A transgenic mouse containing both the *CEACAM5* and *CEACAM6* genes in a large (187 kb) piece of human genomic dna (the CEABAC mouse) has recently been constructed 43. At low-to-moderate expression levels of *CEACAM5* or *CEACAM6* (or both), a partial block in cell differentiation, a mild-to-moderate colonocyte hyperproliferation, and an inhibition of anoikis or apoptosis are evident in the transgenic colon. These mice are found to be significantly more prone to develop carcinogen-induced colon tumours, specifically the traditional adenomatous type 25. At higher (tumour-like) expression levels, a complete block in cell differentiation and extreme colonocyte hyperproliferation can be observed. These mice show massively enlarged colons comprising continuous non-focal cytologic and architectural abnormalities, including dysplastic features and serrated morphology. These results suggest that, although moderate expression levels of *CEACAM5* and *CEACAM6* can cause an imbalance of tissue homeostasis leading to increased tumour susceptibility following the classical pathway of colonic neoplasia, tumour-like expression levels alone produce a severe imbalance leading directly to tumour formation, specifically the serrated subtype. Hence, we propose that *CEACAM5* and *CEACAM6* can play a significant role in both neoplastic pathways (Chan *et al.* Colorectal hyperplasia and dysplasia due to human *CEA* and *CEACAM6* expression in transgenic mice. Submitted manuscript).

## CONCLUSION

*CEACAM5* and *CEACAM6* are commonly considered inert tumour markers, despite the discovery and documentation of their tumorigenic functions over the past two decades. Nevertheless, because of their ectopic or deregulated overexpression in up to 70% of all tumours, *CEACAM5* and *CEACAM6* represent popular targets for novel cancer therapies, including cancer vaccines, cellular immunotherapy, radioimmunotherapy, and antibody therapy. With growing knowledge of the effects of *CEACAM5* and *CEACAM6* on tumour biology, novel therapeutic strategies that focus more on perturbing the tumorigenic functions of these antigens may now be indicated.
